# Machine learning in GI endoscopy: practical guidance in how to interpret a novel field

**DOI:** 10.1136/gutjnl-2019-320466

**Published:** 2020-05-11

**Authors:** Fons van der Sommen, Jeroen de Groof, Maarten Struyvenberg, Joost van der Putten, Tim Boers, Kiki Fockens, Erik J Schoon, Wouter Curvers, Peter de With, Yuichi Mori, Michael Byrne, Jacques J G H M Bergman

**Affiliations:** 1 Department of Electrical Engineering, VCA Group, University of Technology Eindhoven, Eindhoven, Noord-Brabant, The Netherlands; 2 Department of Gastroenterology and Hepatology, Amsterdam UMC—Locatie AMC, Amsterdam, North Holland, The Netherlands; 3 Department of Gastroenterology and Hepatology, Catharina Hospital, Eindhoven, The Netherlands; 4 Digestive Disease Center, Showa University Northern Yokohama Hospital, Yokohama, Kanagawa, Japan; 5 Division of Gastroenterology, Vancouver General Hospital, The University of British Columbia, Vancouver, British Columbia, Canada

**Keywords:** endoscopy, gastrointesinal endoscopy, computerised image analysis

## Abstract

There has been a vast increase in GI literature focused on the use of machine learning in endoscopy. The relative novelty of this field poses a challenge for reviewers and readers of GI journals. To appreciate scientific quality and novelty of machine learning studies, understanding of the technical basis and commonly used techniques is required. Clinicians often lack this technical background, while machine learning experts may be unfamiliar with clinical relevance and implications for daily practice. Therefore, there is an increasing need for a multidisciplinary, international evaluation on how to perform high-quality machine learning research in endoscopy. This review aims to provide guidance for readers and reviewers of peer-reviewed GI journals to allow critical appraisal of the most relevant quality requirements of machine learning studies. The paper provides an overview of common trends and their potential pitfalls and proposes comprehensive quality requirements in six overarching themes: terminology, data, algorithm description, experimental setup, interpretation of results and machine learning in clinical practice.

## INTRODUCTION

Over the last couple of decades, the quality of endoscopic imaging in gastroenterology has increased dramatically. All current state-of-the-art endoscopy systems are equipped with high-definition white light endoscopy (HD-WLE) and preprocessing optical chromoscopy techniques. As a result, the diagnostic challenge in endoscopy has shifted from visualisation to *interpretation*. This paradigm shift, in combination with increasing computational power of modern-day computers, has cleared the way for the application of *machine learning* in endoscopy to aid the endoscopist in the interpretation of these high-quality, multimodality images. In several medical domains, such as radiology and pathology, the use of machine learning has already shown promising results.^1–5^ Recently, there has been a vast increase in machine learning in endoscopic literature.^6–12^ The introduction of deep learning with artificial neural networks has fuelled this increase even further.^13^ Although deep learning offers a powerful tool for machine learning, its application is associated with pitfalls. The relative novelty of this field and an increasing number of machine learning studies pose a challenge for reviewers and readers of endoscopy GI journals, since the quality of reported studies varies significantly.^14^ To appreciate scientific quality and novelty of machine learning studies, understanding of the technical basis and commonly used techniques is required. Clinicians often lack this technical background, while machine learning experts may be unfamiliar with the clinical relevance and implications for daily practice. This review aims to guide reviewers and readers alike of peer-reviewed GI journals in how to interpret machine learning studies in endoscopy and to allow for critical appraisal of the most relevant quality requirements of these studies.

We will first explain the most relevant universal aspects of machine learning in endoscopy. We focus on common trends and their potential pitfalls, and subsequently propose corresponding basic quality requirements. This is clustered into six overarching themes: terminology, data, algorithm description, experimental setup, interpretation of results and machine learning in clinical practice.

## Terminology: the basis of clear communication

Applying the complex technical science of machine learning in the field of clinical endoscopy may lead to terminology, which is prone to misinterpretation, ambiguity and confusion. The collaboration between scientists from engineering and medicine, each with their own jargon and expertise, easily leads to a communication gap. This disconnection is apparent in many publications on machine learning in endoscopy, as the parts describing the clinical setup of the study and the technical background of the algorithm often feel completely disconnected. In this section, we provide a basis for clear interdisciplinary communication by defining the most relevant technical terminology at a conceptual level for readers of endoscopic GI journals, summarised in table 1. A more extensive explanation of commonly used technical terminology, yet not addressed in this review, can be found in table 2.

**Table 1:** Overview of the most commonly used terminology in machine learning literature (addressed in this review)

Machine learning	The use of mathematical models for capturing structure in data. After the optimisation procedure on example data—so-called training—the models can be used to make predictions about new, unseen data.
Features	Visual properties of the data that are quantitatively summarised in an array of numbers. In conventional machine learning, these features are clinically inspired and thus handcrafted, while in deep learning these features are automatically learnt from the data.
Computer-aided detection (CADe)	Machine learning algorithms applied to medical data for primary detection of pathology (eg, polyp detection).
Computer-aided diagnosis(CADx)	Machine learning algorithms applied to medical data for predicting diagnoses (eg, polyp classification).
Deep learning	A form of machine learning in which a neural network of several layers is used, exploiting hierarchical relations in the data. The major difference from conventional machine learning is that these features and relations are all learnt from the data, a property which is also referred to as *end-to-end learning*.
Pretraining	Training a deep learning algorithm with data that are different from the target data. This technique can be exploited to first train a rough model on a large set that can be fine-tuned using a smaller dataset of interest. ImageNet is by far the most commonly used dataset for pretraining.
Transfer learning	This is used after a deep neural network is pretrained on a large dataset that is different from the target data. Generally, a dataset is used with general imagery not specific to the final purpose of the algorithm. This pretrained model extracts basic discriminating features from the large dataset and these features and their weights are then ‘transferred’ for training and fine-tuning on new data which are specific for the target purpose of the model—often applied when sufficient target data are lacking to train the network from scratch.
Hyperparameters	Almost all machine learning models are regulated by so-called hyperparameters, which govern the model architecture and its training procedure. Examples of common hyperparameters in neural networks are the number of layers and the learning rate. These parameters can generally not be optimised during the training process and are typically chosen based on a number of trials using empirically driven approach.
Hyperparameter optimisation	The process of finding the right hyperparameters of a model, based on the performance on the validation set. This is performed either by using a grid-search, in which a number of options are defined for each hyperparameter and all combinations are systematically evaluated, or using a random search, in which the values are randomly sampled from a predefined range.
Ensemble learning	Instead of training a single model on the whole dataset, one can also train multiple models that are trained slightly differently to yield a prediction about the same data point. These models are generally trained on different subsets of the data and with slightly different hyperparameters. Averaging the scores of different models generally leads to a better and more robust prediction.
Training dataset	A set of data (examples) on which the mathematical model is optimised (trained). In supervised learning, the examples are labelled, and the model is trained to predict the labels of the samples.
Validation dataset	A separate set of data samples that can be used to tune the hyperparameters of the model. A model can be trained several times with different hyperparameter values (on the training set) and the ones that achieve the best performance on the validation set are chosen. Often referred to as ‘internal validation’.
Test dataset	A set of data samples neither used for training the model nor for optimisation of the hyperparameters. The performance on the test set reflects how good the model generalises to new, unseen data.
Cross-validation	A validation approach that is more robust to outliers than a regular hold-out approach. In K-fold cross-validation, the data used for training and validation are split into K parts, after which the model is subsequently trained with K-1 folds of data and validated on the left-out fold. This is repeated for all folds after which the scores are pooled.
Overfitting	A phenomenon that occurs when the model is too tightly fitted to the training data and does not generalise to new data (ie, the model only works for the given training examples). Overfitting can be recognised by high training performance combined with low test performance.
Data augmentation	A way to artificially enhance the size of a dataset, by adding slightly distorted copies of the original data points to the training set. The samples are distorted in such a way that the labels do not change after applying the transformation (eg, rotation, slight skewing, minor zooming, adding noise). The use of data augmentation generally leads to more robust models.

**Table 2:** Overview of commonly used terminology in machine learning literature, not further described in this paper

Support vector machine (SVM)	An efficient machine learning algorithm that aims to find a line (hyperplane) separating data with maximum margin between two classes. SVMs can be linear or non-linear; the latter is more powerful, but also more prone to overfitting.
Random forests	An ensemble machine learning algorithm in which a large amount of binary decision trees are trained, each using a different (random) subset of the data (ie, bagging) and with different split options (sampled randomly).
Backpropagation	A method for training neural networks, in which the network is first used to make a prediction for a given sample, after which the error is propagated back through the network for updating the network weights such that the error will be reduced. This is repeated many times for all the data points in the dataset, until the network is said to *converge* and the error does not significantly decrease anymore.
Regularisation	A collection of techniques that can be used to counter overfitting. This can be done either by explicitly introducing some model constraints in the mathematical optimisation procedure (training) or implicitly, for example, by using slightly distorted copies of the data during training (also known as data augmentation).
Batch normalisation	A method to force the network activations of each layer into a certain range, so that the network can optimally learn from errors during backpropagation.
Gradient descent	A mathematical optimisation procedure in which the gradient of a function is exploited to move towards a (local) minimum of a function. In machine learning this is generally the loss function, which captures the number of errors the model makes. The gradient is used to subsequently take steps on this function along the steepest slope downwards.
Mini-batch	A group of data samples for which the loss is jointly computed during backpropagation in order to make an update step. Mini-batches are generally sampled randomly without replacement. Once there are no data points left, one epoch is passed (see epoch).
Epoch	During backpropagation, all data points pass through the network either individually or in mini-batches in order to update the model and minimise the loss/error. An epoch represents the period for which all data points have passed through the network once.
Learning rate	During the training of a neural network, the model gradually adjusts its weights until the prediction error on the data is minimised. The direction of these updates is determined by gradient descent optimisation, while the magnitude of these updates is governed by the learning rate. A large learning rate will lead to fast convergence towards an optimum, but if the update steps are too large, the real optimum can never be achieved.
Classification	Classification is a form of supervised learning, for which the input comprises numerical data (eg, images) and the goal of the algorithm is to match that input with a target class of a predefined set of potential categories at the output. An example here would be polyp classification, where a polyp can be either hyperplastic, sessile serrated or adenomatous.
Regression	Regression is a form of supervised learning, for which the input comprises numerical data (eg, images) and the goal of the algorithm is to match that input with a target continuous numerical value at the output. For example, estimating the oxygen saturation of the blood based on an image of the mucosa.
Object detection	Object detection is a form of supervised learning, for which the input comprises numerical data (eg, images) and the goal of the algorithm is to detect whether or not an object from a predefined list of objects is present in that image and indicate its location within the image, typically with a rectangular bounding box, at the output. An example is polyp detection in colonoscopy.
Image segmentation	Image segmentation is a form of supervised learning, for which the input comprises an image and the goal of the algorithm is to segment parts of that image that are associated with a predefined category or set of categories at the output. Typically, the output is numerical mask, indicating for each pixel to what category it belongs to. An example is lesion segmentation in Barrett’s oesophagus.

### Commonly (mis-)used terminology

#### Machine learning

The field of machine learning aims to build mathematical models based on given data that have predictive power about new, unseen data. When the given data are provided with a certain label, for example ‘dysplastic’ or ‘non-dysplastic’, this is called *supervised* learning, and when there are no (gold standard) labels associated with the data, this is called *unsupervised* learning. While supervised learning aims to predict the labels of new data points based on a model learnt from labelled examples, unsupervised learning aims only to find the underlying structure of the data, for example, to predict what data points are similar. This latter aspect can be helpful when a gold standard is not available (eg, due to the size of the dataset), or in cases where there is no obvious gold standard and one is looking only to split the data points into meaningful groups that share certain properties. The popular term ‘Artificial Intelligence’ (AI) is commonly used interchangeably with ‘machine learning’, but it addresses a much broader field that also includes reasoning and natural language processing. Roughly speaking, while machine learning can only be applied for specific tasks, AI aims to develop a more generic form of autonomous learning.

#### Features

Before a machine learning algorithm generates a prediction, the input data are summarised in a compact representation, which is generally an array of numbers. This representation expresses the properties of the data that help separate different classes or clusters. The numbers in this representation are referred to as features. For example, when separating lemons from oranges, the shape and the colour of the fruits can be expressed in numbers, which serve as useful features to separate the two classes. In conventional machine learning, these features are typically selected by the investigators based on human knowledge of the particular field. Modern-day deep-learning approaches, in contrast, learn the best features automatically based on large sets of data.

#### Deep learning

While often presented as an alternative to machine learning, deep learning is actually a form of machine learning, in which a deep (artificial) neural network (DNN) is employed. Loosely inspired by the mammalian brain, a DNN consists of several layers of densely interconnected artificial neurons. As in biological neurons, each artificial neuron receives a weighted input from the neurons in the previous layers and ‘fires’ when a certain threshold is exceeded. As each neuron responds to a different pattern, a layer of the network will work as a step of abstraction, starting at raw pixels of an image at the input of the network and ending at a class label at the output. Neurons in the first layers will respond to basic features, such as points and edges, which are combined into simple shapes by the next layer and used by the layer after that to construct objects, finally causing neurons in the deeper layers to fire if a certain combination of objects is present in the image, leading to a prediction about the class of the image. In this way, a hierarchical representation of the input data is obtained by the network: pixel contrast differences make edges, edges make basic shapes (eg, circles, lines, rectangles), shapes make objects (eg, a nose, mouth and eyes) and objects lead to a class prediction (eg, a face). While artificial neural networks have been introduced decades ago, breakthrough results were achieved only recently, when researchers invented techniques that are crucial for training deeper networks, that is, having a larger number of layers. The depth of these networks turns out to be crucial for capturing the complex relations that are present within an image.

#### Transfer learning and pretraining

Training deep neural networks generally requires a lot of labelled data (ie, at least thousands of samples). This amount of data is not always available for specific machine learning tasks, in particular for medical applications. A method called transfer learning alleviates this problem by first training the network on a large set of data for which labels are readily available (eg, ImageNet^15^) (ImageNet is a publicly available dataset of 1.2 million labelled images, containing 1000 object categories such as ‘pillow’, ‘flamingo’ and ‘syringe’) and subsequently exploiting the learnt network for the target classification task (eg, lesion classification). This can be achieved using two different approaches: (1) using the entire network as a feature extractor (see also *features*) without further optimising the network and then train a simple classification method using those features (also referred to as CNN codes) or (2) optimising the parameters of the network by retraining some of the layers in the network using the target data.^16^ The optimal strategy depends on the size of the dataset for the target problem and its similarity to the data that was used for pretraining. The initial training on a different dataset is called pretraining and it allows the network to already learn generic patterns and structures that are also useful for the target classification problem.

#### Hyperparameters

Almost all machine learning methods have a number of optional settings that are defined by the investigator. These settings are referred to as hyperparameters and affect the behaviour of a model and can be used to optimise its performance. This is comparable to the settings on a photo camera, where you can manually set the exposure time and the aperture, thereby changing the picture quality under varying conditions. Generally, two types of hyperparameters are relevant for neural networks: (1) model hyperparameters, defining the architecture of the model (eg, number and type of layers) and (2) training hyperparameters that determine the training process (eg, the learning rate and type of regularisation).

#### Computer-aided detection and computer-aided diagnosis

Machine learning algorithms can be applied to assist in the *interpretation* of medical imagery, often referred to as computer-aided detection (CADe) and computer-aided diagnosis (CADx). An important distinction between CADe and CADx algorithms is that the first are developed to primarily *detect* pathology, while CADx algorithms are designed to *classify* pathology (ie, a CADe detection algorithm red flags a colonic polyp; a CADx algorithm classifies it as adenomatous or hyperplastic). Finally, machine learning algorithms can be applied to guide interventions, usually referred to as *image-guided interventions,* for example, when an algorithm detects a lesion and helps to guide a biopsy needle using ultrasound imaging. In this paper, we focus on CADe and CADx algorithms, since these represent the vast majority of publications in endoscopic literature.

#### Training and overfitting

**Figure 1:**
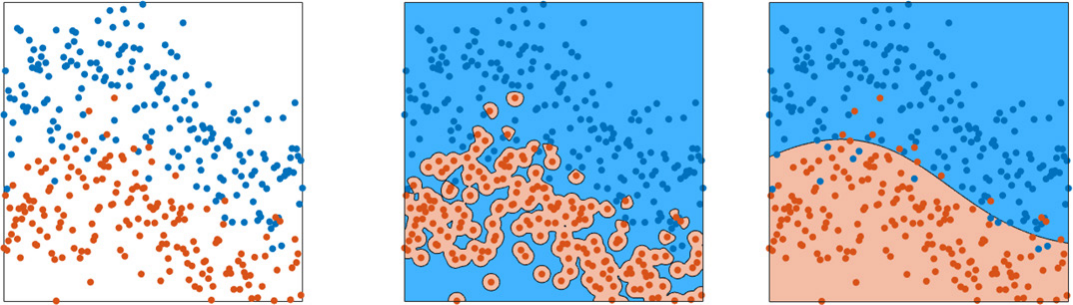
Graphical display of overfitting of training data. In this figure, the leftmost panel displays data points of two classes, in which the class is indicated by the colour. The centre panel shows the same data including the prediction of a model trained on that data as the background colour. Overfitting is clearly visible as the model isolates points of the red class, rather than capturing the class as a whole. The rightmost panel shows the prediction of a different model as background colour. Although this model makes mistakes (red points can be seen on a blue background and vice versa), this model demonstrates better generalisation, as it captures the class distributions rather than individual points.

Different mathematical machine learning models exist to describe the relation between an input (eg, image) and a desired output (eg, a label). Support vector machines, random forests or neural networks are popular model choices. Although different machine learning models are constructed in different ways, all of them use data for building the model. Building such a model based on data is referred to as training, since the model learns from given examples. During training, driven by mathematical optimisation methods, a model will gradually improve in capturing the input-output relation of the given examples in the training data. For example, given a set of images of polyps and the associated class of these polyps, it will learn to predict the class, based on the image. Once a model is trained, it should also work for new data, which was not used for training the model. This property is called generalisation. If the model only works for the training data, but does not work well for new data, this is called *overfitting,* as the model is too tightly fitted to the training data and does not generalise towards new data (figure 1). Especially for smaller sets and more complex models, overfitting poses a serious problem.

#### Training, validation and test sets

**Figure 2:**
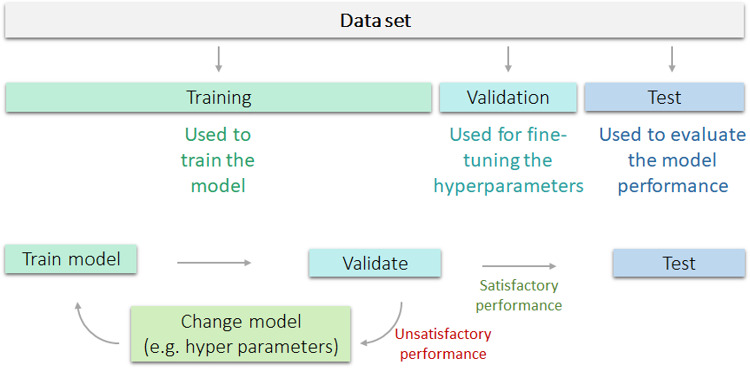
Visualisation of training, validation and test set and overfitting, and their appropriate use. The training dataset is used to train the model, followed by validation. In case of unsatisfactory performance, the model is changed, retrained and again validated. In case of satisfactory performance, the model is then tested on a separate test set to evaluate model performance.

In building a machine learning model in endoscopy, the available data are generally split into three distinct sets: (1) training set, (2) validation set and (3) test set (figure 2). The training set is used to build the model (eg, to train a model that predicts a label based on image features). To check that the model is not overfitted to the training data, the validation set is used. Using this separate set, one can verify that the model also works for unseen data. In addition, almost all machine learning models are in part regulated by so-called hyperparameters, for which optimal choices can also be selected based on the performance on the validation set. Finally, a third part is used to evaluate the model that is trained with the optimal hyperparameters. This part of the data is referred to as the test set. It is important that the hyperparameters are not selected based on the model’s performance on the *test set*, as this would bias the model towards the unseen data and thus create a type of overfitting. This effect is also known as *data leakage,* since data that should be used only to test performance are also used to optimise the model.

#### Cross-validation

**Figure 3:**
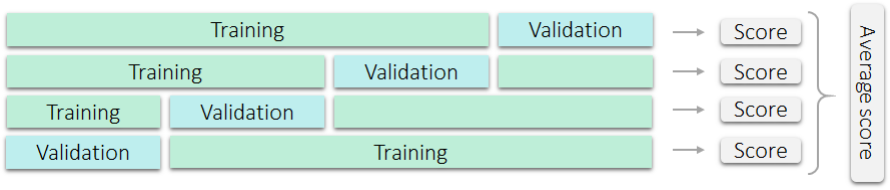
Graphical display of fourfold cross-validation.

As the availability of annotated endoscopic data is often limited for training and validating CAD systems, a specific validation procedure can be used to optimally exploit the available data, which is called cross-validation. In this validation approach, data are split into a number of equal parts, most often four, after which one part is used as a validation set, while the remaining (in this case three) parts are used for training. This is repeated a total of four times, each time with a different validation set, after which the scores of the four experiments are pooled into a point estimate, yielding a result that is more robust against data variation. Figure 3 graphically displays a dataset with fourfold cross-validation. When using cross-validation, it is important that the partitioning of the data is performed on a patient basis: data from a single patient can only occur in one of the four parts. When this approach is not carefully followed, cross-validation will likely provide a biassed overestimate of the algorithm performance. For example, when researchers present cross-validation results where different polyps from the same patient are included in multiple ‘folds’ (ie, both in training and validation parts), this will likely lead to an overestimation of the model performance. A good way to deal with an unbalanced dataset, for example, by including many polyps from a single patient versus only a small number of polyps from other patients, is to stratify the dataset in size and class balance. Note that, after optimising the hyperparameters (figure 2) using cross-validation, still, an independent test set is necessary to estimate the algorithm performance.

## Data: the fuel of machine learning algorithms

Machine learning algorithms, in particular those using *deep learning* techniques, rely heavily on the availability of large annotated datasets. Large-scale, high-quality acquisition of representative data is however challenging, since both quantity and quality of datasets are important for optimal performance, regardless of algorithm structure. Below we describe several frequent occurring pitfalls.

### Selection bias, overfitting and representativeness of data

It is important to realise that machine learning algorithms will pick up on any discriminative feature that allows them to better separate the data, irrespective of whether this discriminative feature is logical, clinically relevant or clearly a result of bias.^13^ A great example is a study in which an algorithm was developed to recognise neoplasia in histology slides. The algorithm appeared to be highly effective at first sight, yet in fact was found to recognise ink marks placed by pathologists to indicate abnormal areas rather than actual neoplastic morphology.

Most machine learning studies in endoscopy have used *retrospectively* collected datasets, often collected in previous studies with strict selection criteria.^17^ These datasets are generally retrieved from single-centre databases in expert centres, containing many similar samples that do not cover the natural variability of imaged objects, endoscopists and imaging devices in daily practice.^18^ These studies are therefore prone to selection bias (ie, the non-random selection of data that does not fully represent the study population and thereby limits the external validity of results) and overfitting, a common phenomenon in machine learning.^13^ Overfitting leads to overestimated results that lack representativeness in a general endoscopic setting. The risk of overfitting is further increased when only static, endoscopic images are used, since endoscopists tend to only capture and store high-quality images. Videos, encompassing 30 video frames per second, often contain a larger variety in image quality (eg, by the presence of blur, stool or partially obscured lesions).^8 10^ Thereby video analysis is likely to add to the robustness of CAD systems, lowering the risk of overfitting, compared with an image-based approach.

A general rule to minimise the risk of overfitting is to use datasets that are both large and heterogeneous. The use of multiple heterogeneous datasets is preferred, since heterogeneous training leads to robustness and is one of the best measures to prevent overfitting. Such datasets should ideally be independent, meaning that they at least exhibit no overlap in terms of patients, but preferably also vary on other aspects such as the operator, medical centre or acquisition protocol. Prospectively collected datasets are less prone to selection bias and are often more similar to a general endoscopic setting. That said, the collection of large prospectively collected datasets is cumbersome. Nevertheless, prospectively collected datasets should be preferred over retrospectively collected datasets in test sets.

To enable identification of bias and overfitting in CAD studies, it is important that all datasets used in the process of training, validation and testing are well defined. A complete description of datasets encompasses *at least*: data source (eg, random search in endoscopic database, existing study cohort, prospective protocol); inclusion and exclusion criteria of imagery; number of collected and excluded images and number of patients. Finally, some basic technical information of imagery and any preprocessing techniques should be stated (eg, resolution, file type).^19^ Moreover, it is important to consider these technical aspects when planning to construct such a dataset, as they can negatively impact the results in a real-time setting and even lead to overfitting. For example, if a lossy compression standard such as JPEG is being used in a multicentre study, it is likely that slightly different compression settings are employed for each centre. An algorithm could exploit this bias to learn that images with a certain compression type are more likely to be of a certain class. This is an especially notorious type of bias, as modern compression standards are optimised to produce artefacts that cannot be perceived by the human visual system. Table 3 shows a complete overview of basic data requirements.

**Table 3:** Checklist of key elements that should be described in machine learning papers, structured by manuscript section

Methods		Page
Data	Minimise risk of overfitting by the use of multiple, heterogeneous and independent datasets.	
	Provide a complete description of the data acquisition process.	
	Describe the basic technical information of the imagery and the use of any preprocessing methods.	
	Define a reliable gold standard for all data used to train, validate and test the model.	
	Algorithms that localise lesions on images and videos, reliable gold standard input for the model should incorporate annotations by multiple experts.	
	Provide detailed information on ethics approval concerning the use of patient data.	
Algorithm architecture	Provide a basic description of the algorithm architecture and clear-cut motivation for the most relevant technical choices.	
	Describe extensive technical details in separate technical publications, or in supplementary materials.	
Experimental set-up	Describe the experimental set-up of the algorithm and choose the appropriate performance metrics.	
	Primary outcome parameters should be based on the envisioned clinical application of the model.	
	Do not optimise hyperparameters on test set.	
	Ensure that training, validation and test sets are always split on a patient-basis.	
	Report a complete overview of all evaluated models to prevent ‘cherry-picking’ of the best performing algorithms.	
Results		
	Results should be presented with caution and in a structured approach.	
Discussion		
	Include a section where data selection bias, overfitting and generalisability are explicitly discussed.	
	Describe the necessary steps towards clinical implementation.	

### Gold standard development

Next to the availability of multiple independent datasets, which should preferably be large and heterogeneous, the reliability of the label of interest is of crucial importance. We can only build a reliable model if relevant predictive features are related to an outcome which is ‘true’. This implies that for all the datasets we are using to build the model (for learning, validation and testing), the label of interest should be unequivocally established. This is called the gold standard (or ground truth) input for the algorithm. For CADx algorithms, the gold standard input is generally the histology of the corresponding image. Such a classification model then allows for image-based prediction of the histology of a new image, for example, normal versus neoplastic, or adenomatous versus hyperplastic polyp.^10 20 21^ It should however be noted that histology might not always be the preferred gold standard for CADx systems, for example, due to sampling error and interobserver variability between pathologists.^22^ For example, in certain circumstances, optical biopsies by experts might be preferred over pathology assessment.

Providing a suitable gold standard for localisation algorithms is more challenging, since these have to classify imagery, and generally have to localise lesions within imagery. This latter constraint holds for most CADe algorithms, therefore they are generally trained with manual annotations. However, for localisation of a lesion within an image, it is nearly impossible to obtain a pixel-precise annotation based on the pathology findings. Therefore, in addition to a histopathological gold standard label of the image, delineations by multiple endoscopists offer a reasonable estimate of the location of the lesion within the image. This is a laborious process, generally performed by expert endoscopists.

**Figure 4:**
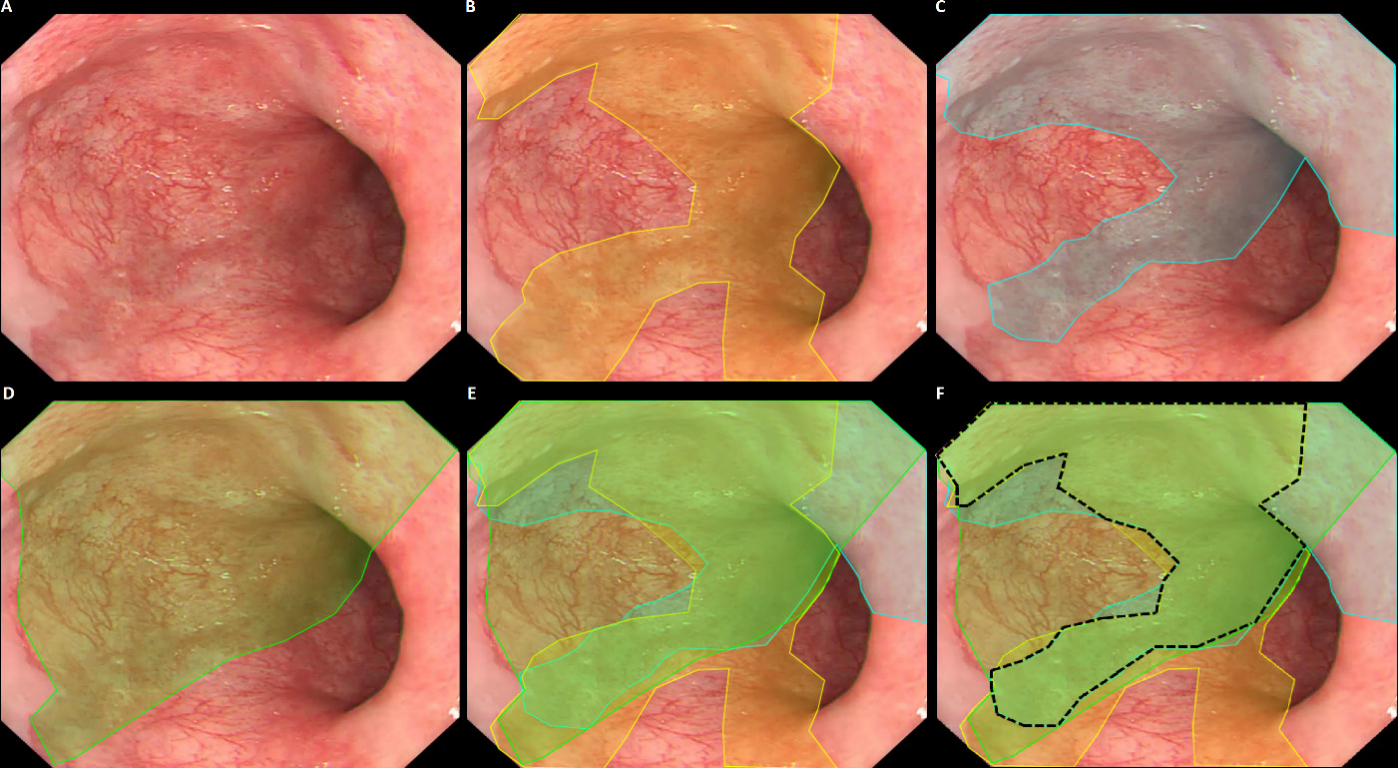
Exemplary case of subtle Barrett’s neoplasia, delineated by three experts (yellow, blue and green). Parts of the lesion (‘the sweet spot’) are recognised by all experts (black), yet other parts are only recognised by one or two experts. Reprinted from Bergman J, de Groof AJ, Pech O, et al. An interactive web-based educational tool improves detection and delineation of Barrett's esophagus-related neoplasia. Gastroenterology 2019;156:1299-1308, with permission from Elsevier.

For this reason, often a single expert annotation is used for training and/or testing.^18 23 24^ It is important to realise that such gold standard annotations are only an approximation of the actual gold standard, that is, the demarcation line of underlying pathology. Since experts tend to disagree on pixel-precise annotations, causing interobserver variability, single expert delineations are subjective and less accurate. By including multiple expert delineations for each image, it is possible to differentiate between ambiguous image areas and areas where there is expert consensus. Especially for targeted biopsies, the expert consensus provides a valuable estimate of the optimal biopsy location. Figure 4 displays an exemplary image of subtle Barrett’s neoplasia for which three experts were asked to indicate the borders of the lesion. Although all three experts agreed on a central part of the lesion (‘the sweet spot’), they disagreed on other parts. The ‘sweet spot’ logically has a higher likelihood to contain neoplasia, and therefore contains more information and thus serves as a better gold standard than the individual expert delineations.^25^ It should be underlined that the localising function of such CAD algorithms only serve to improve the primary detection of neoplastic lesions in overview, allowing targeted biopsies by non-expert endoscopists who otherwise would have missed the lesion. An exact histologically correlated delineation is not required since the actual resection of the lesion will be done using a different endoscopic approach (eg, optical chromoendoscopy with optical magnification), often in another endoscopic session and by a more experienced endoscopist.

In addition to assessing the credibility of the gold standard predictions, exploiting interobserver variability is a growing topic of interest within the machine learning community and could help in making more robust models.^26^

The use of multiple expert annotations per case is therefore preferred in establishing a gold standard for CADe algorithms. To minimise interobserver variability of such gold standard annotations, the annotation instructions for the experts should be determined in advance to avoid a scenario whereby one expert annotates only the gross abnormal part of the lesion yet the other expert attempts to precisely delineate the more subtle periphery of the lesion. In publications, the quality of the gold standard annotations should be clearly described (number of experts, their qualifications and their pre-delineation instructions).

Delineation of videos, containing thousands of video frames, is often performed by non-experts. It is imperative that these delineations are confirmed by experts to ensure accurate and correct gold standard establishment.

### Data ownership

The use of clinical patient data for development of (commercial) CAD systems is currently under debate in the world of AI, involving both ethical and regulatory issues. This debate exceeds the field of machine learning in endoscopy but is of vital importance, since all research groups that are using clinical patient data without specific consent will be confronted with local and international legislation when they are seeking commercialisation of their product. There currently is an unmet need for uniform interpretation of legislation on this topic.^27^ Pending a commonly accepted legal interpretation of this issue, authors should provide detailed information on ethics approval in their scientific papers.

Key messages

- Minimise risk of overfitting by the use of multiple, preferably independent, datasets which should ideally be large and heterogeneous.
- Provide a complete description of the data acquisition process: this allows for assessment of potential selection bias. Insist on an explicit discussion of selection bias and overfitting in the limitations section of all publications.
- Describe the basic technical information of the imagery and the use of any preprocessing methods.
- Define a reliable gold standard for all data used to train, validate and test the model.
- For CADe algorithms, that is, algorithms that localise lesions on images and videos, reliable gold standard input for the model should incorporate annotations by multiple experts.
- There currently is an unmet need for uniform interpretation of legislation on data ownership in the field of AI. Authors should be encouraged to provide detailed information on ethics approval concerning the use of patient data in scientific papers.

## Algorithm description: relevance versus completeness

As mentioned in the previous paragraph, there are many different approaches to construct a machine learning algorithm. For the target audience of GI journals, some insight into the motivation for the choice of the algorithm architecture is vital for a basic understanding of the study. However, interpretation of the technical background of algorithm architectures is challenging. Many studies present extensive technical details in their methodology, thereby overwhelming clinicians with technical terminology which they cannot interpret. In a recent review by Lipton *et al*, the authors suggest that this trend might be caused by the desire to convince reviewers of the technical depth of a study.^28^ A complete technical in-depth description of the algorithm and its novelty is important to allow adequate peer review and to inform readers with a technical background. Therefore, we feel that authors should include the full technical details as supplementary material to the clinical publication. Alternatively, when a previous technical publication exists with a complete technical description of the system, authors should refer this work for the technical details, while addressing the most important elements in the clinical paper.

For a good estimation of readability for the clinical target audience, iterations with clinical coauthors are essential. Technical authors should be encouraged to consult with clinicians prior to publication. As a rule of thumb, for each technical term a short explanatory description should be given to provide insight for the target audience. The readability of clinical papers can, however, also be compromised by reviewers with a technical background. They sometimes tend to request specific technical details that are not appropriate for the main text of a clinical paper. GI journals should therefore pay extra attention to preservation of readability of CAD papers by instructing both their authors and reviewers.

Key messages

- Provide a basic description of the algorithm architecture and clear-cut motivation for the most relevant technical choices.
- Describe extensive technical details in separate technical publications, or in supplementary materials.
- Consult with clinical coauthors to ensure readability of the paper.

## Experimental set-up: generalisability is key

In machine learning, the outcome of a study depends on the amount and quality of data, and on the experimental design of the model. In many publications, the most important methodological limitations relate to inappropriate choices regarding the model’s experimental design. In this respect, three issues are most important.

### The chosen performance metrics and their motivation

There are different ways to express the performance of a model. For the different machine learning tasks, several well-defined metrics have been described: *sensitivity, specificity* and *area under the curve* for two-class classifications, *confusion matrices* and *mean average precision* for multiclass classifications and *intersection over union* (IoU) or the *DICE coefficient* for segmentation (ie, delineation).^29^ However, sometimes these metrics do not fully reflect the desired algorithm outcome. For example, when considerable interobserver variability is present around the borders of gold standard annotations, these areas may be considered of lesser importance than the area for which there is consensus, as described in the previous paragraph. In that case, it is not obvious to apply IoU or DICE, as there are multiple segmentations that are correct, and these metrics can only deal with a single gold standard segmentation per image. In that case, one can use a slightly adapted version of these metrics^25 30^ or aim to explicitly model the interobserver variability and incorporate this into the evaluation metric.^31^ When deviating from the set of default metrics, it is important to provide a clear rationale, to avoid metrics being presented just because they yielded the most favourable results, which would then lead to selection bias and favour overfitting.

### Approach for validation and testing

Another crucial parameter in the experimental design of machine learning algorithms is the chosen approach for validation and testing. As mentioned, machine learning models typically contain several hyperparameters, which can be used to optimise its performance. To test the trained model’s performance, generally a separate test set is used, containing data that not have been used for training the model. While running the trained model on this test set, it may be tempting to adjust the hyperparameters of the existing model based on its performance on the test set. This leads to a form of overfitting generally known as ‘data leakage’ (as information contained in the test data is actually used (ie, ‘leaks’) to train the model instead of only to test its performance). Data leakage thereby often leads to a misrepresentation and overestimation of the model’s actual performance.

To avoid data leakage, usually a validation set is used as an intermediate step in the experimental design. This validation step is then used for optimising the hyperparameters of the model, and thereby preventing data leakage from the test set. In this ‘training set-validation set-test set sequence’, the training set is used to optimise the model’s performance in predicting the gold standard of the training set images, the validation set is then used to optimise the model’s performance in predicting the gold standard of validation set images by optimising the hyperparameters of the model and the test set is used to relate the model’s overall performance in predicting the gold standard of a new dataset to which the model has not yet been exposed.

Finding the right trade-off between the relative shares of these three distinct datasets can be challenging. First, sufficient data are necessary to train a good model: the model should ‘see’ enough examples to ensure that the model can adequately predict the outcome of interest. Second, the validation set should be large enough to find the right hyperparameters and to prevent overfitting. Finally, the test set should be sufficiently large and heterogeneous to reflect the model’s performance in ‘real-life’, with enough statistical power for a meaningful estimation of performance metrics. This latter constraint is most important for to warrant the validity of the results and should never be compromised in favour of having more training examples. Ideally, multiple independent test sets are employed to assess the model’s robustness against different medical centres, acquisition protocols or patient ethnicities.

In addition, the balance between the respective class sizes should be considered carefully. Most papers report performance on a uniform class distribution (eg, 50% of cases with neoplastic lesions and 50% negative controls), while this is hardly ever the case in a real clinical setting, where the incidence of disease is generally much lower than 50%. Training with an approximately uniform class distribution is motivated by preventing the model to develop a bias towards either of the classes. However, researchers should report the expected class probabilities in a clinical setting and infer what their numbers from a uniform test setup would imply in the envisioned application. Table 4 shows an illustrative example of what happens for a system with a good performance in terms of sensitivity and specificity when the incidence drops, resulting a flood of spurious detections that can considerably dilute the number of true detections.

**Table 4:** The expected number of false positives (FPs) per true positive (TP) for a fixed performance and varying incidence

Sensitivity/Specificity	Incidence	#FPs per TP
0.90/0.90	1:1	0.1
	1:10	1
	1:100	11
	1:1000	111
	1:10 000	1111

In most machine learning studies in endoscopy, data acquisition is more driven by data availability and convenience than by the optimal experimental design. This often leads to the use of a single database which is manually split into a training set and validation set, with the test set lacking or also originating from the same single database. Such a situation carries important limitations. First, because the three subsets used for training, validation and testing are derived from the same dataset, these subsets will be homogeneous, which will lead to good performance of the model when carried across the three sets. However, the overall dataset will likely suffer from selection bias and may not reflect the real-life exposure and natural variability. Second, the homogeneity of the three sets will not allow potential overfitting in the training phase or overfitting by hyperparameter optimisation in the validation phase to be picked up. The three datasets will have the same background noise, which will not directly be recognised but will be carried forward throughout the training-validation-test sets as relevant information for the model. Third, a manual split for creating a training set and an internal validation set from a single dataset may create subsets that are not independent. This holds especially if multiple images are derived from the same patient (eg, 800 images obtained from 50 patients) and subsets are created by dividing images instead of patients. In such instances, the training set and validation set will contain different images but stem from the same patient. Such images are clearly not independent, and the validation set will generate results that are too optimistic since they reflect overfitting. Therefore, the optimal experimental design of a machine learning model in endoscopy incorporates a training phase, a validation phase and a testing phase. Ideally, these should consist of independent datasets and not originate from a manual split of the same database.

### Involved hyperparameters and method to determine their optimal values

The third crucial factor in the experimental design of machine learning algorithms relates to multiple testing of different models and cherry-picking of only the ‘best performing algorithms’. During the early phases of algorithm development, choices are made regarding the general model architectures, for example, one can use different CNNs, different ensemble learning techniques and different ways of cross-validation. While this is good practice at these early phases, during which it helps to select the most promising candidates, care should be taken not to redesign the architecture at later phases of algorithm development. When testing a large number of models, it is very likely that one model will yield good results on the test set. A common trend is that authors only select the best performing algorithms, and disregard the other models (ie, ‘cherry-picking’). A similar effect occurs when authors describe a cross-validation procedure, in which several models are trained and evaluated (one for each cross-validation fold), and subsequently continue their analysis only on the best performing fold. This is contradictory, since the rationale of cross-validation is to average out such outliers rather than to highlight them.

Key messages

- Clearly describe the experimental set-up of the algorithm and choose the appropriate performance metrics.
- Be aware of the risk of overfitting when splitting datasets and do not optimise hyperparameters on the test set.
- Ensure that training, validation and test sets are always split on a patient-basis.
- Report a complete overview of all evaluated models to prevent ‘cherry-picking’ of the best performing algorithms.

## The art of interpreting results

Correct interpretation of results in machine learning is challenging because of its multidisciplinary nature. While acknowledging the fact that virtually all studies will be limited by at least some form of selection bias and overfitting, the general advice to authors is to be cautious when interpreting results and presenting conclusions.

Results should be interpreted both in the light of the limitations of experimental design of the model and the quality and generalisability of the datasets used, and the model’s envisioned clinical application. For example, a CAD system designed for detection of early Barrett’s neoplasia should be tested on test sets with subtle lesions that are hard to detect by endoscopists. Testing the CAD system with datasets containing obvious lesions might lead to high accuracy, but represents less clinical usefulness. A well-reasoned and structured approach is recommended. This starts with a logical order in the presentation of results, based on preset outcome measures. These outcome measures (or the model’s performance parameters) should be based on the envisioned application in clinical practice, and preferably benchmarked with endoscopist performance. This is crucial to assess if and to what extent the CAD system will be of beneficial value to the endoscopist. When developing a CAD system for primary detection of colonic polyps, the system should be tested on videos without focus on any specific areas of interest, mimicking daily practice during scope withdrawal, where a polyp might be missed. Testing such a system on dedicated videos of polyps, however, is not the envisioned application in daily practice, since a primary detection system by definition is not focused on any abnormalities. Technical scientists tend to perform a variety of experiments to assess the general performance of an algorithm, even when experiments and performance parameters deviate from a logical clinical application. A driving force for this phenomenon is that technical science journals tend to focus on the implementation of technical novelty in machine learning, rather than to value its potential clinical relevance. Yet, in a clinical paper these experiments merely distract readers from assessing the value in clinical practice. This again highlights the importance of having continuous multidisciplinary iterations in scientific collaborations, to ensure that the right message is conveyed to the target audience.

Especially in early stage research, using small, retrospectively collected datasets can make it challenging to extrapolate results to a clinical setting, and results should be interpreted with caution. In that respect, authors should acknowledge these limitations and describe the envisioned steps towards clinical implementation, including the challenges they expect to face in this process.

Key messages

- In general, results should be presented with caution and in a structured approach.
- Primary outcome parameters should be based on the envisioned clinical application of the model.
- All machine learning papers should include a section where limitations regarding data selection bias, overfitting and generalisability are explicitly discussed.
- The necessary steps towards clinical implementation should be clearly described.

## The role of machine learning in clinical practice

What is the future role of machine learning in endoscopy? Currently, the most commonly applied machine learning systems in endoscopy are focused on lesion detection (CADe) and characterisation (CADx). Most studies focus on colonic polyps, small bowel bleeding foci, gastric cancer and oesophageal cancer. Recently, an increasing number of studies describe the use of video analysis and its potential advantages over the use of still images. Video-based algorithms have several theoretical advantages over image-based algorithms. As a video is basically a set of still images over time, it contains spatiotemporal information that is not available when using individual still images. By incorporating such spatiotemporal relationships CAD performance may be improved: for example, two sequential video frames with almost overlapping spatial predictions more likely represents a neoplastic lesion than two sequential video frames where the spatial predictions have no overlap. However, a video-based approach may not necessarily be superior to an image-based system. This holds especially for detection of lesions that are relatively subtle. For example, early neoplastic lesions in Barrett’s oesophagus are difficult to detect endoscopically: they are associated with less apparent morphological changes than other CAD targets such as colonic polyps or angiodysplasias of the small bowel. In addition, Barrett’s lesions occur against a background mucosa that much more resembles the neoplastic abnormality than the normal colonic mucosa or small bowel mucosa do for colonic polyps and angiodysplasias, respectively. Optimal endoscopic visualisation of Barrett’s oesophagus therefore requires optimal image quality by a combination of adequate insufflation, clean mucosa and no blurring due to motility, breathing and patient movement. During real-time inspection of Barrett’s oesophagus, most expert endoscopists inspect the Barrett’s segment with video endoscopy and then strive to obtain an optimal still image in overview, by sequential freezing/unfreezing the video sequence, thereby disregarding images of suboptimal quality, until an optimal still image is acquired. When the endoscopist is asked to capture only video input for a Barrett’s CAD algorithm, these quality constraints cannot be guaranteed, potentially leading to (1) missed lesions due to insufficient video quality and (2) spurious detections distracting the endoscopist. Moreover, whereas the endoscopist would actively control image acquisition when capturing optimal still images for an image-based algorithm, a video-based alternative may give the false impression that a simple video recording of the Barrett’s segment will suffice. As a consequence, the algorithm will be supplied with inferior quality information compared with a still image-based approach.

Multiple commercial parties, including the three leading endoscopy manufacturers, and many renowned research groups, are currently developing CAD systems. Some of these parties have already presented prototype systems, such as the WavSTAT4 optical biopsy system (Pentax Medical), EndoBRAIN system (Olympus), GI Genius intelligent endoscopy module (MedTronic) and the NvisionVLE system (NinePoint Medical). These are all introduced as ‘game-changers’ in the field of endoscopy, yet studies reporting results of CAD implementation during real-time procedures are still scarce. This shows that the bench-to-bedside development is a complex process that requires strong multidisciplinary collaborations.

Commercial parties, however, in their desire to protect their intellectual property, will most likely produce ‘black-box’ systems, without supporting scientific evidence. This is a phenomenon comparable to the introduction of optical chromoscopy over a decade ago: a conceptually interesting and promising technique is introduced, yet with little scientific evidence prior to commercial launch. This is partly driven by the anticipation that thresholds for clinical implementation of many CAD systems will not be high, due to the fact that most of these systems rely on a ‘low risk, high impact’ principle, although currently regulatory entities do not necessarily consider AI to be low risk.^32^ A spurious algorithm prediction will at worst lead to an additional biopsy, yet the algorithm may detect a cancer that might otherwise have been missed. Furthermore, nearly all CAD systems are now considered as second-readers, merely assisting endoscopists.^33^ However, this may lead to an abundance of poorly tested commercialised CAD systems, negatively affecting the general credibility of the technology. On the other hand, if we wait for perfection, we will be waiting forever and we would be unnecessarily denying patients the best possible care at that point in time. Hence, CAD systems that have been tested thoroughly in an ex vivo setting, should be allowed for in vivo scientific testing by regulatory bodies, to further assess which tools are truly useful in clinical practice. This should preferably be done in international multicentre studies with sufficient statistical power (including a sample size calculation), to enable estimation of clinical performance and robustness of the CAD system.

How should the leading endoscopy societies deal with these developments? Before clinical implementation, ideally all CAD systems should be well-tested through controlled clinical trials. Second, in order to objectively evaluate performance and enable direct comparison between CAD systems, heterogeneous test datasets may be collected by endoscopy societies, on which CAD systems can be tested for performance thresholds to clinical implementation. Such benchmarking datasets should ideally meet several requirements. They should contain heterogeneous data, representing the natural variability in terms of lesion appearance and quality. Second, they should contain an adequate sample size. Third, such datasets would be designed for measurement of algorithm performance, yet should not be available for training of candidate algorithms.

Endoscopy societies should play a leading role by providing guidance and establishment of quality requirements. The first step could be initiated by the designation of a consortium of international key opinion leaders in the field of machine learning in endoscopy, to reach consensus on evidence-based, minimum quality standards for reporting machine learning papers. This could subsequently lead to a formal international guideline, similar to the Consolidated Standards of Reporting Trials (CONSORT) guidelines or TRIPOD statements.^34 35^

It is worth mentioning that in endoscopy we should critically appraise the additive value of all new diagnostic tools, and their pitfalls. There already is a significant problem with performance of redundant gastrointestinal endoscopies. New diagnostic tools may exacerbate this overutilisation. For example, CAD systems that enable primary detection of colonic polyps might also lead to redundant resection of many clinically irrelevant and harmless hyperplastic polyps, increasing the length of endoscopic procedures, medical costs and patient burden. These considerations are often neglected during evaluation of new diagnostic tools, but are highly relevant. This highlights the importance of preset outcome measures that are based on the envisioned clinical application.

CAD algorithms can only detect what is shown to them by the endoscopist. Although this has not been extensively interrogated, machine learning systems might therefore also aid in improving or monitoring endoscopic quality.^36^ These quality assurance (QA) algorithms might serve as a ‘referee’ for endoscopic quality standards, for example, indicating how much colonic surface area is missed during a pullback, when the mucosal surface needs to be cleaned, or colonic withdrawal speed needs to be slowed down. The argument could be made that this might have a larger impact on clinical outcomes than a detection tool for specific gastrointestinal pathology. QA algorithms are therefore likely to increasingly become a field of interest in endoscopy.

Key messages

- Most research in machine learning in endoscopy is currently focused on detection and characterisation.
- Studies reporting implementation of clinically relevant improved outcome of CAD in daily endoscopic practice are rare.
- Endoscopy societies should play a leading role in establishing quality requirements for CAD in clinical practice.
- New machine learning tools should be critically appraised, balancing potential clinical value versus overuse of endoscopy.
- Quality control in endoscopy is a promising potential application of machine learning.

## Conclusion

Machine learning has the potential to revolutionise the field of endoscopy. In recent years, there has been a rapid increase in the use of machine learning in endoscopic literature. This has led to an unmet need for a multidisciplinary evaluation of quality requirements in machine learning research in endoscopy. In this paper, we have initiated this evaluation, by providing insight into several key aspects of machine learning.
